# Supplementary material for: SARS-CoV-2 Proteins Bind to Hemoglobin and Its Metabolites
Source: Int J Mol Sci. 2021 Aug 21;22(16):9035. doi: 10.3390/ijms22169035 (PMC8396565; doi:10.3390/ijms22169035)
Supplement: Supplementary file 1 [file ijms-22-09035-s001.zip › ijms-1285593-supplementary.pdf]

HEME binding proteins (PDB Code)	Hemoglobin human (Uniprot Code)	Nucleoprotein Sars-CoV (Uniprot Code)
1a00:A	P02008	NCAP_SARS
1a3o:A	P02042	A0A679GC99_SARS2
1bz1:A	P02100	A0A6H2EFU6_SARS2
1bzz:A	P69892	A0A6G8I2S4_SARS2
1c0h:A	P68871	A0A6H1PLH4_SARS2
1c7d:A	P69905	NCAP_SARS2
1dhb:A	Q9NZD4	A0A6C0T6Z7_SARS2
1dsh:A	P09105	A0A6C0N5E8_SARS2
1fhj:A	Q6B0K9	A0A6H2L5M8_SARS2
1hbr:A	Q1W6G9	A0A6B9VLF5_SARS2
1hda:A	A0A1K0GXZ1	A0A6C0WXA2_SARS2
1hds:A	D1MGQ2	A0A6H0MAP2_SARS2
1ibe:A	A0A1K0GUV5	A0A6H1PIQ7_SARS2
1j7s:A	U6A216	
1mhb:A	A0A2R8Y7C0	
1ns6:A	A0A1S5UZ39	
1o1k:A	A0A385HVZ2	
1o1l:A	A0A385HW21	
1o1m:A	A0A385HW02	
1o1n:A	Q6J1Z9	
1o1o:A	Q86YQ1	
1o1p:A	A0A385HVY8	
1pgh:A	A0A385HW00	
1s0h:A	A0A0K2BMD8	
1v75:A	Q86YL2	
1xy0:A	I1VZV6	
1xye:A	Q9BX83	
1xz5:A	E9M4D4	
1xz7:A	Q9NQT3	
1xzu:A	Q96T46	
1xzv:A	E1B2D1	
1y09:A	Q86YQ4	
1y0a:A	E9LUX2	
1y0c:A	V9H1D9	
1z8u:B	P78461	
2dhb:A	Q4ZGM8	
2mhb:A		
2pgh:A		
2qls:A		
2qsp:A		
2qu0:A		
2ri4:A		
2zfb:A		
2zlt:A		
2zlu:A		
3a59:A		
3at5:A		
3cy5:A		
3d4x:A		
3dht:A		
3eok:A		

3gdj:A
3gou:A
3gys:A

The search returned 399 sequences with motifs HEME binding, but only 68 structures present complexes only with the HEME group.

Protein	Residue	Complex ASA[b]	Monomer ASA[b]	Pair Potential	Hotspot Status [a]	Hotregion Status	Complex ASA[b]	Monomer ASA[b]
HB_A	LEU35	26.04	73.04	17.36	NH		46.51	130.4
HB_A	PRO38	42.98	61.77	2.43	NH		58.51	84.09
HB_A	LYS41	0	0	13.92	NH		0	0
HB_A	THR42	0	0	0	NH		0	0
HB_A	PRO45	0	0	4.99	NH		0	0
HB_A	HIS46	0	0	9.17	NH		0	0
HB_A	PHE47	0	0	14.04	NH		0	0
HB_A	ASP48	0	0	19.97	H	2	0	0
HB_A	SER50	0	0	17.74	NH		0	0
HB_A	HIS51	0	0	19.4	H	-	0	0
HB_A	GLY52	0	0	11.09	NH		0	0
HB_A	SER53	0	0	7.25	NH		0	0
HB_A	ALA54	0	0	7.56	NH		0	0
HB_A	GLN55	0	0	12.46	NH		0	0
RBD_C	GLU340	47.25	67.63	7.68	NH		81.38	116.5
RBD_C	ALA344	0	0	7.56	NH		0	0
RBD_C	THR345	0	0	6.17	NH		0	0
RBD_C	ARG346	0	0	27.67	H	2	0	0
RBD_C	PHE347	0	0	22.7	H	2	0	0
RBD_C	ALA348	0	0	17.73	NH		0	0
RBD_C	SER349	0	0	12.03	NH		0	0
RBD_C	TYR351	0	0	24.76	H	-	0	0
RBD_C	ALA352	0	0	16.19	NH		0	0

RBD_C	TRP353	0	0	16.59	NH		0	0
RBD_C	ASN354	0	0	10.77	NH		0	0
RBD_C	ARG355	0	0	15.53	NH		0	0
RBD_C	ASN450	0	0	13.12	NH		0	0
RBD_C	TYR451	0	0	31.74	H	1	0	0
RBD_C	LEU452	0	0	33.83	H	1	0	0
RBD_C	ARG466	26.46	58.18	12.34	NH		63.18	138.9
RBD_C	ILE468	22.33	56.43	10.14	NH		39.11	98.82
RBD_C	LEU492	0	0	22.69	H	1	0	0

[a] NH denotes non hot spot and H denotes hot spot and [b] Solvent Accessible Surface Area (ASA)

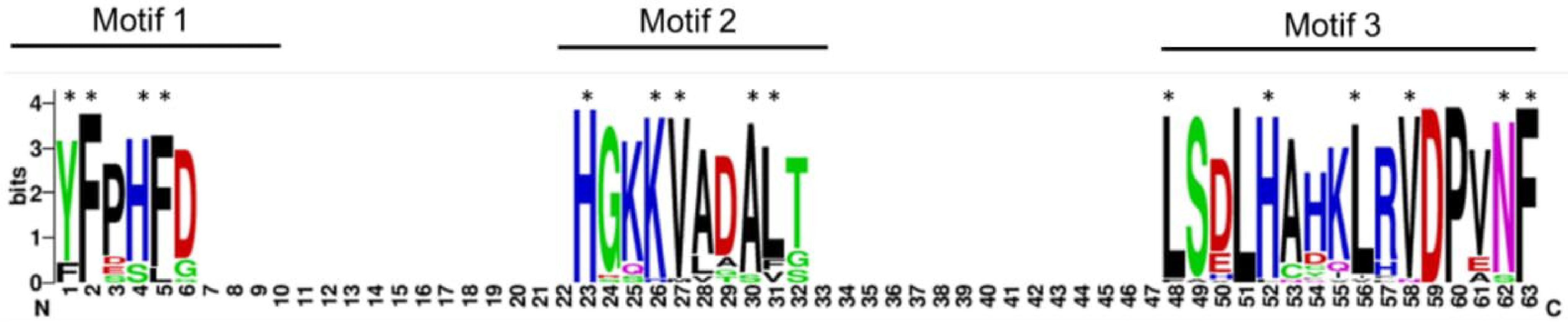

Motifs	p-value	q-value
YFPHFD	1.2×10^{-12}	3.7×10^{-6}
HGKKVADALT	4.2×10^{-13}	1.3×10^{-6}
LSDLHAHKLRVDPVNF	8.4×10^{-21}	$2.5x10^{-14}$

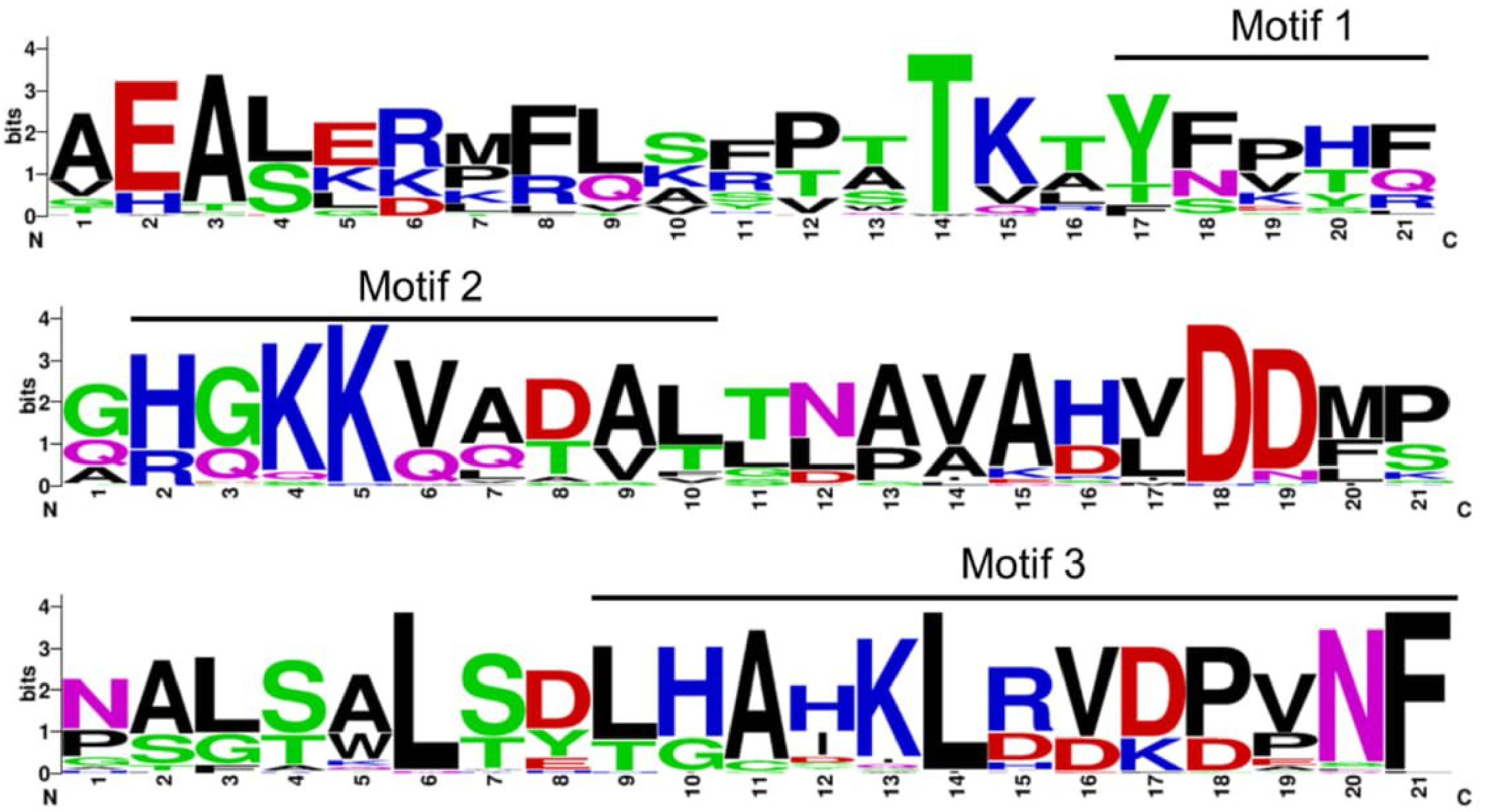
Motif 1
Motif 2
Motif 3
bits
N
C

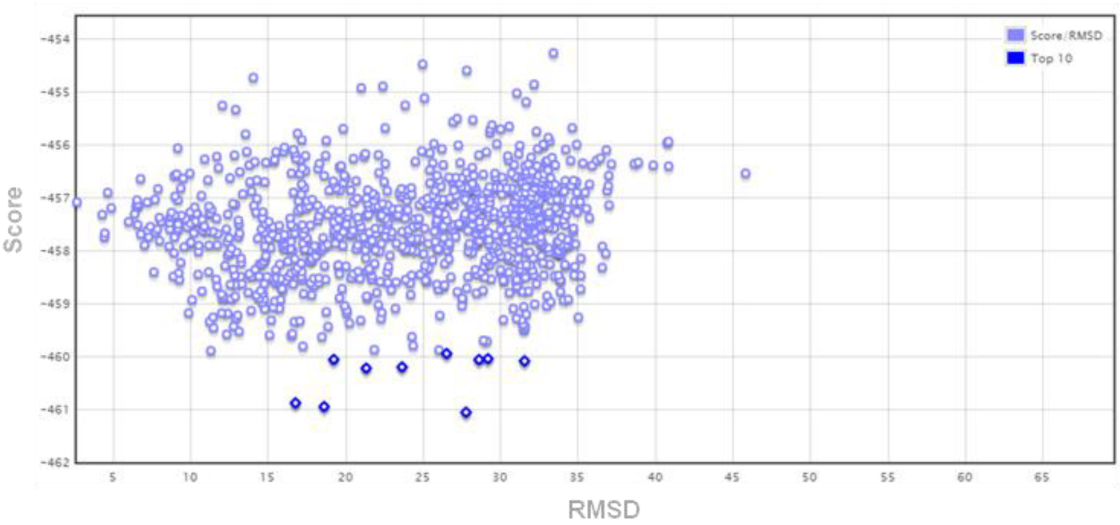
Score/RMSD
Top 10
-454
-455
-456
-457
-458
-459
-460
-461
-462
Score
5
10
15
20
25
30
35
40
45
50
55
60
65
RMSD
